# Supplementary material for: Modeling glioblastoma heterogeneity as a dynamic network of cell states
Source: Mol Syst Biol. 2021 Sep 16;17(9):e10105. doi: 10.15252/msb.202010105 (PMC8444284; doi:10.15252/msb.202010105)
Supplement: Supplementary file 6 — Source Data for Figure 5 [file MSB-17-e10105-s004.zip › Figure5A_sourcedata/GSEA_3071/nosigdb_stateV.GseaPreranked.1622795159954/SEURAT_CELLCYCLE.html]

Details for gene set SEURAT\_CELLCYCLE[GSEA]

|  || Dataset | state43071 |
| Phenotype | NoPhenotypeAvailable |
| Upregulated in class | na\_pos |
| GeneSet | SEURAT\_CELLCYCLE |
| Enrichment Score (ES) | 0.7954428 |
| Normalized Enrichment Score (NES) | 4.3032975 |
| Nominal p-value | 0.0 |
| FDR q-value | 0.0 |
| FWER p-Value | 0.0 |
Table: GSEA Results Summary

  

Fig 1: Enrichment plot: SEURAT\_CELLCYCLE      
 Profile of the Running ES Score & Positions of GeneSet Members on the Rank Ordered List

  

| PROBE | GENE SYMBOL | GENE\_TITLE | RANK IN GENE LIST | RANK METRIC SCORE | RUNNING ES | CORE ENRICHMENT || 1 | ATAD2 |  |  | 2 | 2.214 | 0.0219 | Yes |
| 2 | HMGB2 |  |  | 5 | 2.011 | 0.0417 | Yes |
| 3 | NUSAP1 |  |  | 6 | 2.001 | 0.0618 | Yes |
| 4 | PCNA |  |  | 10 | 1.908 | 0.0804 | Yes |
| 5 | TOP2A |  |  | 14 | 1.842 | 0.0983 | Yes |
| 6 | MCM4 |  |  | 17 | 1.815 | 0.1161 | Yes |
| 7 | RAD51AP1 |  |  | 22 | 1.757 | 0.1330 | Yes |
| 8 | CENPF |  |  | 28 | 1.705 | 0.1492 | Yes |
| 9 | TYMS |  |  | 30 | 1.683 | 0.1659 | Yes |
| 10 | CCNE2 |  |  | 32 | 1.664 | 0.1824 | Yes |
| 11 | RRM2 |  |  | 34 | 1.660 | 0.1989 | Yes |
| 12 | MKI67 |  |  | 45 | 1.598 | 0.2130 | Yes |
| 13 | HELLS |  |  | 48 | 1.588 | 0.2286 | Yes |
| 14 | GINS2 |  |  | 57 | 1.528 | 0.2424 | Yes |
| 15 | DTL |  |  | 60 | 1.503 | 0.2571 | Yes |
| 16 | GTSE1 |  |  | 66 | 1.491 | 0.2711 | Yes |
| 17 | E2F8 |  |  | 67 | 1.488 | 0.2860 | Yes |
| 18 | CDK1 |  |  | 69 | 1.488 | 0.3008 | Yes |
| 19 | CLSPN |  |  | 84 | 1.417 | 0.3123 | Yes |
| 20 | CDCA2 |  |  | 96 | 1.382 | 0.3241 | Yes |
| 21 | RRM1 |  |  | 99 | 1.364 | 0.3374 | Yes |
| 22 | POLD3 |  |  | 111 | 1.340 | 0.3488 | Yes |
| 23 | TACC3 |  |  | 116 | 1.334 | 0.3614 | Yes |
| 24 | SMC4 |  |  | 127 | 1.320 | 0.3727 | Yes |
| 25 | FEN1 |  |  | 139 | 1.296 | 0.3836 | Yes |
| 26 | GMNN |  |  | 146 | 1.280 | 0.3953 | Yes |
| 27 | NCAPD2 |  |  | 157 | 1.252 | 0.4060 | Yes |
| 28 | TMPO |  |  | 161 | 1.243 | 0.4179 | Yes |
| 29 | KIF23 |  |  | 167 | 1.235 | 0.4293 | Yes |
| 30 | CDCA3 |  |  | 169 | 1.230 | 0.4415 | Yes |
| 31 | NASP |  |  | 180 | 1.212 | 0.4518 | Yes |
| 32 | NUF2 |  |  | 192 | 1.189 | 0.4616 | Yes |
| 33 | USP1 |  |  | 202 | 1.168 | 0.4716 | Yes |
| 34 | UBE2C |  |  | 214 | 1.154 | 0.4811 | Yes |
| 35 | CDC45 |  |  | 228 | 1.138 | 0.4900 | Yes |
| 36 | MCM2 |  |  | 238 | 1.130 | 0.4996 | Yes |
| 37 | NDC80 |  |  | 240 | 1.129 | 0.5108 | Yes |
| 38 | MCM5 |  |  | 243 | 1.127 | 0.5217 | Yes |
| 39 | TPX2 |  |  | 248 | 1.122 | 0.5322 | Yes |
| 40 | BRIP1 |  |  | 249 | 1.121 | 0.5434 | Yes |
| 41 | BUB1 |  |  | 277 | 1.084 | 0.5491 | Yes |
| 42 | MCM6 |  |  | 282 | 1.080 | 0.5592 | Yes |
| 43 | CKAP2L |  |  | 285 | 1.078 | 0.5697 | Yes |
| 44 | PRIM1 |  |  | 293 | 1.074 | 0.5791 | Yes |
| 45 | ECT2 |  |  | 295 | 1.071 | 0.5897 | Yes |
| 46 | AURKB |  |  | 297 | 1.068 | 0.6002 | Yes |
| 47 | CKAP2 |  |  | 312 | 1.053 | 0.6081 | Yes |
| 48 | CDC6 |  |  | 328 | 1.040 | 0.6156 | Yes |
| 49 | CDCA7 |  |  | 330 | 1.038 | 0.6259 | Yes |
| 50 | RFC2 |  |  | 337 | 1.025 | 0.6350 | Yes |
| 51 | WDR76 |  |  | 350 | 1.015 | 0.6429 | Yes |
| 52 | EXO1 |  |  | 354 | 1.010 | 0.6525 | Yes |
| 53 | KIF11 |  |  | 369 | 0.982 | 0.6597 | Yes |
| 54 | CASP8AP2 |  |  | 379 | 0.970 | 0.6677 | Yes |
| 55 | TTK |  |  | 383 | 0.966 | 0.6768 | Yes |
| 56 | UHRF1 |  |  | 390 | 0.958 | 0.6853 | Yes |
| 57 | SLBP |  |  | 403 | 0.949 | 0.6925 | Yes |
| 58 | HJURP |  |  | 406 | 0.945 | 0.7016 | Yes |
| 59 | DLGAP5 |  |  | 418 | 0.936 | 0.7089 | Yes |
| 60 | UBR7 |  |  | 423 | 0.932 | 0.7175 | Yes |
| 61 | DSCC1 |  |  | 427 | 0.926 | 0.7262 | Yes |
| 62 | CENPE |  |  | 437 | 0.918 | 0.7337 | Yes |
| 63 | LBR |  |  | 438 | 0.916 | 0.7429 | Yes |
| 64 | POLA1 |  |  | 463 | 0.900 | 0.7473 | Yes |
| 65 | BLM |  |  | 486 | 0.879 | 0.7519 | Yes |
| 66 | RAD51 |  |  | 522 | 0.851 | 0.7537 | Yes |
| 67 | BIRC5 |  |  | 541 | 0.842 | 0.7587 | Yes |
| 68 | HMMR |  |  | 622 | 0.788 | 0.7513 | Yes |
| 69 | CENPA |  |  | 627 | 0.786 | 0.7584 | Yes |
| 70 | CDC25C |  |  | 684 | 0.755 | 0.7552 | Yes |
| 71 | GAS2L3 |  |  | 685 | 0.755 | 0.7628 | Yes |
| 72 | UNG |  |  | 703 | 0.745 | 0.7670 | Yes |
| 73 | ANP32E |  |  | 722 | 0.739 | 0.7710 | Yes |
| 74 | CTCF |  |  | 729 | 0.731 | 0.7772 | Yes |
| 75 | CKAP5 |  |  | 752 | 0.723 | 0.7802 | Yes |
| 76 | AURKA |  |  | 761 | 0.718 | 0.7859 | Yes |
| 77 | CBX5 |  |  | 783 | 0.708 | 0.7889 | Yes |
| 78 | PSRC1 |  |  | 787 | 0.705 | 0.7954 | Yes |
| 79 | NEK2 |  |  | 901 | 0.659 | 0.7803 | No |
| 80 | KIF20B |  |  | 934 | 0.647 | 0.7807 | No |
| 81 | CKS2 |  |  | 966 | 0.634 | 0.7811 | No |
| 82 | CDCA8 |  |  | 1043 | 0.610 | 0.7726 | No |
| 83 | MSH2 |  |  | 1107 | 0.590 | 0.7664 | No |
| 84 | KIF2C |  |  | 1309 | 0.530 | 0.7331 | No |
| 85 | CCNB2 |  |  | 1373 | 0.513 | 0.7262 | No |
| 86 | CKS1B |  |  | 1502 | 0.479 | 0.7064 | No |
| 87 | G2E3 |  |  | 1602 | 0.459 | 0.6920 | No |
| 88 | TUBB4B |  |  | 1659 | 0.444 | 0.6857 | No |
| 89 | ANLN |  |  | 2125 | 0.371 | 0.6001 | No |
| 90 | RPA2 |  |  | 2570 | 0.314 | 0.5179 | No |
| 91 | RANGAP1 |  |  | 3006 | 0.268 | 0.4370 | No |
| 92 | CHAF1B |  |  | 3047 | 0.264 | 0.4320 | No |
Table: GSEA details [plain text format]

  

Fig 2: SEURAT\_CELLCYCLE: Random ES distribution      
 Gene set null distribution of ES for **SEURAT\_CELLCYCLE**
